# Researches upon the Necropolis of New Orleans, with Brief Allusions to Its Vital Arithmetic

**Published:** 1851-02

**Authors:** 


*Researches upon the Necropolis of New Orleans, with brief allusions to its Vital Arithmetic.* By BENNET DOWLER, M.D., of New Orleans.

We are indebted to the courtesy of Dr. Dowler for a copy of his paper on the Necropolis of New Orleans, in advance of the regular issue of the New Orleans Medical and Surgical Journal, and the pressure of other matter upon this Journal has prevented an earlier acknowledgment of Dr. Dowler's politeness.

The investigation of the vital statistics of a community is one of the most useful and valuable employments of the medical mind, and its proper management requires mental qualifications of a high and peculiar kind. There are so many circumstances that must necessarily be constantly before the mind, and they are often so intimately blended, and so frequently impenetrate each other, that the powers of analysis are often at fault. Yet they must be separated, each particular portion must have its due bearing, and each series or group, must be restrained within its proper boundary. Even with all the aids that governments sometimes give to the subject of vital statistics, its proper management is one of great difficulty. Even in the midst of the greatest apparent fulness of material, the mind strains after essential points of knowledge that have not yet been attained. The vital statist of one hundred years ago, would have considered himself overburdened with riches, if he had possessed the materials that are now open to the uses of vital statistics, but the statist of the present, constantly finds his subject stretching beyond his materials into the regions of obscurity.

With a full knowledge of all of the inherent difficulties of his subject Dr. Dowler determined to abandon the ordinary paths of investigation, and turned his attention from the figures of the living to the revealments of the city of the dead. It was a bold and original thought, but in the hands of Dr. Dowler it has assumed life, harmony, and strength. The voice of the tomb does not utter, altogether, an uncertain sound upon these subjects; it may speak expressively of the sanitary history of its climate, and should not be neglected, even in the use of other materials of vital statistics.

We have read the paper on the Necrology of New Orleans with a high degree of satisfaction, but it is impossible to make an intelligible abstract of its facts. The various elements of the material are so intimately interwoven that it is impossible to cut any part without disturbing the integrity of the whole. But we think Dr. Dowler amply sustains the fact that New Orleans has a much higher reputation for sickness than it is fairly entitled to. The following remarks, forming the peroration of Dr. Dowler's article, present his well-sustained conclusions:

"The scope of this paper does not include the special investigation of the climate of New Orleans as it affects immigrants alone. Acclimation is a subject so difficult, so extensive and so important, that it cannot be disposed of in a summary manner. Accurate, popularized information on this subject, is a great desideratum, and might prevent the senseless sacrifice of hundreds of lives, which ignorant, avaricious heads of families make, almost every year, for the hope of gaining dollars. Of the vast number of destitute families who come from high northern latitudes to this city, few comparatively, intend to settle permanently, and few remain beyond the acclimating period. Hence arises a useless waste of life. The acclimated are constantly replaced by the unacclimated. Wave follows wave—epidemic, epidemic. The parent, who, for the mere chance of gain, without intending to become a permanent resident, brings his family from a cold climate to New Orleans, during the hot season, is not guiltless; nor is the writer who would conceal from the immigrant the real dangers of southern acclimation, altogether innocent. The prudent who possess means to command the physical comforts, including the timely aid of doctors and nurses, incur but little danger during an epidemic, and obtain great advantages in their subsequent immunity from epidemic fevers. Accurate knowledge of the climate, is one of the greatest means of obviating its temporary ill effects upon the immigrant, during acclimation, after which, as Shakspere saith,

> "The blessed Gods
> Purge all infection from our air, whilst you
> Do climate here."

The vital perturbations of New Orleans, in modern times, have been unexampled. Its great epidemic, causes the population to vibrate to and fro, with almost the regularity of a pendulum. This, it must be admitted, is presumptive proof against the healthfulness of the climate for strangers.

"Many circumstances, in no way connected with the salubrity of New Orleans, combine to produce every year, an ebb and flow, in its population. The maturation and transportation of the crops of the great Valley of the Mississippi, the navigable condition of its numerous rivers, the concentration of vessels from sea, and the mildness of the climate from November to July, all conspire to attract a multitude to the city during a portion of the year, only, making New Orleans a grand caravansary, a commercial panorama, a lottery for speculation, as well as a statistical enigma, not to mention the social evils inherent to a transitional, if not a chaotic condition of society. Besides all this, the emigration of the acclimated, repress the progress and paralyzes the energies of the city, vitally, socially, and commercially. Immigration alone, keeps alive the yellow fever, which, but too often, fills the streets with hearses, and blackens the reputation of the city for salubrity.

"A French philosopher declares, that there is always something in the misfortunes of others, which doth not displease us. Without sanctioning this maxim, the people of New Orleans may, nevertheless, comfort themselves with the reflection that they live, on an average, much longer than the citizens of New York, taking the Report of the Board of Health for authority. This same authority, which pronounces London to be "twice as salubrious as New Orleans," shows that during last year, the average age at death, was, with the trifling exception of three months, equal to that of London.

"The Report shows that the mortality of New Orleans from consumption and all other pulmonary diseases is, about half as great as in Philadelphia, New York, Havana, Baltimore, and Charleston; sometimes much less, and sometimes a little more than this ratio: thus, omitting fractions, in every 100 deaths in the two cities first named, 28 die of these diseases, while in New Orleans, the proportion is only 13; and, for consumption alone, only 9; being not half as great as in Havana, where it is 19. This statement, in connection with another from the same source, showing that the average life of New Orleans is much longer than in the northern cities, proves that the former, will compare with the latter, most favorably, as it regards salubrity and long life. This favorable comparison will become more apparent, as soon as the sociological equilibrium shall be duly established between the dynamical and statical elements of the population of New Orleans."

Dr. Dowler has performed a very acceptable service by the investigations in the Necropolis of New Orleans, of which we speak, and we hope that he will find time for the prosecution of inquiries upon the kindred themes to which he refers in the admirable paper before us. They are of the highest importance to the philosophy of medicine, and it would afford us sincere pleasure to record Dr. Dowler's triumphs over the difficulties of those subjects. B.

